# Modeling transitions in body composition: the approach to steady state for anthropometric measures and physiological functions in the Minnesota human starvation study

**DOI:** 10.1186/1476-5918-7-16

**Published:** 2008-10-07

**Authors:** James L Hargrove, Grete Heinz, Otto Heinz

**Affiliations:** 1 Department of Foods and Nutrition, University of Georgia, Dawson Hall, Athens, GA, USA 30602; 2 24710 Upper Trail Drive, Carmel, CA, USA 93923

## Abstract

**Background:**

This study evaluated whether the changes in several anthropometric and functional measures during caloric restriction combined with walking and treadmill exercise would fit a simple model of approach to steady state (a plateau) that can be solved using spreadsheet software (Microsoft Excel^®^). We hypothesized that transitions in waist girth and several body compartments would fit a simple exponential model that approaches a stable steady-state.

**Methods:**

The model (an equation) was applied to outcomes reported in the Minnesota starvation experiment using Microsoft Excel's Solver^® ^function to derive rate parameters (k) and projected steady state values. However, data for most end-points were available only at t = 0, 12 and 24 weeks of caloric restriction. Therefore, we derived 2 new equations that enable model solutions to be calculated from 3 equally spaced data points.

**Results:**

For the group of male subjects in the Minnesota study, body mass declined with a first order rate constant of about 0.079 wk^-1^. The fractional rate of loss of fat free mass, which includes components that remained almost constant during starvation, was 0.064 wk^-1^, compared to a rate of loss of fat mass of 0.103 wk^-1^. The rate of loss of abdominal fat, as exemplified by the change in the waist girth, was 0.213 wk^-1^.

On average, 0.77 kg was lost per cm of waist girth. Other girths showed rates of loss between 0.085 and 0.131 wk^-1^. Resting energy expenditure (REE) declined at 0.131 wk^-1^. Changes in heart volume, hand strength, work capacity and N excretion showed rates of loss in the same range. The group of 32 subjects was close to steady state or had already reached steady state for the variables under consideration at the end of semi-starvation.

**Conclusion:**

When energy intake is changed to new, relatively constant levels, while physical activity is maintained, changes in several anthropometric and physiological measures can be modeled as an exponential approach to steady state using software that is widely available. The 3 point method for parameter estimation provides a criterion for testing whether change in a variable can be usefully modelled with exponential kinetics within the time range for which data are available.

## Background

The present article will: 1) Explain how to use a widely available spreadsheet to fit data for time-dependent changes in body composition and function to an exponential model of approach to steady state; 2) Indicate the degree to which changes in waist girth (an important index of risk for the cardiovascular metabolic syndrome) fit the model; and 3) Ask whether the model also describes changes in a variety of anthropometric, physiological and biochemical functions using data from the Minnesota human starvation experiment [1]. As implemented in a spreadsheet, the model could be used for data logging for individuals or groups.

Even though compartmental models of human body composition are widely used, most models are static and the question of whether dynamic transitions are linear, exponential or mixed is not commonly addressed. Kleiber [2] indicated that the rate at which body mass is lost during starvation in humans (([3] and dogs [4] fit curves described by a single exponential function better than it fit a linear rate of loss. A mono-exponential model of change in body mass has been reported [5], but a bi-exponential (2 compartment) model with fat mass (FM) and fat free mass (FFM) also fits the data for patients undergoing weight loss after gastric surgery [6]. In principle, models (equations) for approach to steady state [7] are symmetrical in the sense that they may be applied during gain or loss of weight or other measures. For example, the rate of increase in FM and FFM during overfeeding in men can also be modeled using a simple exponential, asymptotic equation [8]. Because the approach to steady state produces an asymptote that would occur during weight loss caused by food restriction (or weight gain caused by overfeeding) alone or combined with exercise training [9], it is sometimes called the plateau principle.

The Minnesota experiment provides an important public data set that is still being used for detailed mathematical modeling to make inferences about changes in body composition and substrate utilization [10-12]. It is a rare case in which the stipulation for a relatively fixed rate of energy input and expenditure was met in a study with humans over a long period. Subjects were required to maintain a moderately high level of physical activity with 22 miles of outdoor walking and half an hour of treadmill exercise per week during 24 weeks of caloric restriction. In order to test the generality of the plateau principle in reference to changes in multiple end points, we have analyzed changes in body composition, anthropometric measurements, and several functional measures that were observed in the Minnesota study on human starvation. In addition to body mass, FM and FFM, changes in BMI, waist girth, limb girths, resting energy expenditure (REE), heart volume, and work capacity were evaluated.

## Methods

### Characteristics of the Minnesota starvation study

The study reduced the energy intake of 32 male conscientious objectors (20–33 y old, mean 25.5 y) to decrease body mass comparably to severely undernourished prisoners of war with the aim of testing methods for rehabilitating starved men. Individual body mass prior to the weight loss program ranged from 62 kg to 83.6 kg (mean = 69.39 ± 5.85 kg) for a mean height of 178.8 ± 6 cm. Body mass index (BMI) ranged from 18.4 to 25.4 kg/m^2 ^(mean of 21.7). Subjects were slightly taller and thinner than Caucasian selective service registrants for 1943 and were in good physical condition.

The study included a 12-week control phase (weeks C1–C12), 24 weeks of energy restriction (weeks S1–S24), and 20 weeks of recovery (R1–R20). During weeks C1–C12, energy intake was adjusted to bring individuals towards the group norm, based on weight for height, with a mean weight loss of 0.80 kg. Physical activity included 22 miles per week of outdoor walking and additional walking on campus, plus custodial duties. All subjects were also required to walk at 3.5 miles per hr for half an hr per week on a motor-driven treadmill with a 10% grade. The control diet contained about 100 g of protein, 400 g of carbohydrates, and 130 g of fat. Energy intake averaged 3,492 kcal/d (14.62 mJ/d) for the last 3 control weeks, during which group weight declined only 0.3 kg. At that time, REE was 1608.5 ± 91.5 kcal/day. Thus total energy intake balanced energy expenditure, and the group's "activity ratio" was about 2.17 (3,492/1608). From then on, subjects were fed at a level that was expected to cause a 24% group average decrease in body mass during the next 24 weeks. Feeding protocols were individualized so that subjects who were underweight for their height initially were expected to have a smaller percent weight loss than heavier subjects.

Weight loss was induced by reducing food intake to two daily meals with 51 g of protein, 286 g of carbohydrates, and 30 g of fat, with 3 basic menus consisting of cereal, whole-wheat bread, potatoes, turnips, and cabbage, supplemented by scant amounts of meat and dairy products. During the first 12 weeks of starvation, mean energy intake was 1621 kcal d^-1^, which fell to an average of 1514 kcal d^-1 ^during the final 12 weeks of starvation. During the entire starvation period, walking 22 miles a week and custodial work remained mandatory. Nevertheless, at the end of the starvation period, the activity ratio had dropped to 1.52 (mean energy intake of 1514 kcal/REE of 994.5 kcal), with weight close to stability.

### Theoretical basis for the weight loss protocol

The percent weight loss goal was determined by the extent to which each subject exceeded or fell short of the weight for height standard, not by initial percent fat. For individual subjects, energy intake was adjusted so that progressive weight loss would plateau as approximated by a parabolic curve according to an equation:

(1) W_x _= W_f _+ K(24 - t)^2^

where the value of K was calculated as:

(2) K = (W_0_/100) × (P/(24^2^))

W_x _is weight expected for week x, W_f _is final weight after losing a specified percentage of initial weight (P). The value of P ranged from 19% to 29%. K is thus an individualized parameter calculated on the basis of a subject's initial body mass, W_0_, in Eq. 2 and of his specific P. The individual constant for weekly weight loss averaged 0.029 and ranged from 0.021 for the most underweight to 0.041 for the heaviest subjects. Weekly weight loss thus differed for each subject, but it proceeded at the same pace over time for every subject. Caloric adjustments were limited to decreased or increased numbers of servings of bread and/or potatoes. Half the total weight loss was to occur by the eighth week and three-quarters by the twelfth week. The desired 19% to 29% weight loss (averaging 24%) was achieved by the end of the 24^th ^week.

### Anthropometric and clinical measurements

**Figure 1:**
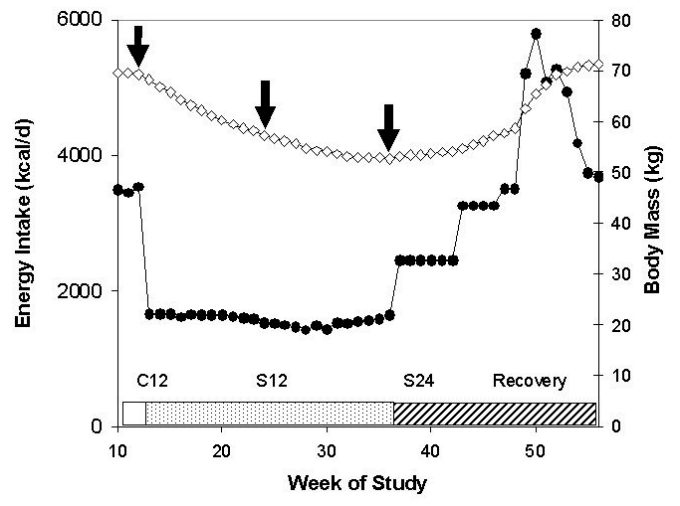
**Protocol for the Minnesota human starvation study**. After a 12 wk control period with balanced energy intake and expenditure, energy intake (closed circles) was reduced to about 1600 kcal/d and adjusted so that the 32 subjects would achieve a 24% loss of body mass (open diamonds) during the next 24 wks. Arrows indicate body mass at weeks C12 (end of control period), and after 12 and 24 weeks (S12 and S24) of energy restriction when most data were collected. During the recovery phase, 4 groups were fed different amounts of energy at increasing levels. The bar at the bottom indicates the control period (unfilled), starvation (hashed) and recovery (stippled).

Except for body mass, most measurements were made only at the end of the 12 week control period (C12) and repeated at weeks 12 (S12) and 24 (S24) of the starvation period (Figure 1). Measurements that held nearly constant included height, skeletal width and depth of the thorax at fifth-rib level, as well as lower trunk width at the iliac crest and tronchanteric level (see [1], Table 48, p. 134, vol. 1). Measurements that changed during starvation and reflected changes in weight included REE, heart volume, the bi-deltoid width, the chest circumference, the waist circumference at the umbilicus, and three limb circumferences: the mid-thigh, the bicep, and the calf. Body composition assessment was made by underwater weighing at the end of the control period and during the 12^th ^and 24^th ^week of starvation. Initial percent body fat averaged 14.0% (6.4 – 26%). Indirect assessment of fat-free mass by urinary nitrogen excretion and estimation of "active tissue mass," that part of the fat-free mass that changed during starvation, was limited to about half the subjects. Changes in performance of selected aerobic and strength tests were indirect indicators of changes in muscle mass. REE was calculated on the basis of the reported basal metabolic rate of oxygen consumption and the reported respiratory quotient.

In order to compare observed waist girths to a standard based on frame size, we adapted a waist reference girth formula [13](girth with normal young-adult fatness) to umbilical waist measurement from Keys' four skeletal measurements at the beginning of starvation: the chest width, chest depth, biiliac width, and bitrochanteric width measurements. A normal reference weight based on these skeletal measurements and stature was adapted from a previously developed formula [14].

### Modeling the approach to steady state during energy restriction

The method of analysis of approach to steady state [15,16] was applied to data from the Minnesota starvation experiment. Data were entered into spreadsheets in Microsoft Excel^® ^2003 and fit to the following equation:

(3) *M*_*t *_= *M*_0 _+ (*M*_*ss *_- *M*_0_)*(1 - *e*^-*kt*^)

M_t _is the value of a dependent variable such as body mass at any time t from 0–24 wks, M_0 _is the initial value, M_ss _is the value (asymptote) at the theoretical new steady state (not at 24 weeks), and k is the apparent first order rate parameter. In [1], measurements were taken weekly and rate units are wk^-1^. In Excel, Equation 1 could be written as follows, with the parameters M_0_, M_ss_, and k in appropriate cells:

(4) = $D$1 + ($D$2 - $D$1)*(1 - EXP(-$D$3*A2))

An Excel file has been provided [see Additional file 1] that shows how to solve Eq. 4, how to perform non-linear curve fitting, and how to obtain kinetic values by the 3 point method described below. A second Excel file [see Additional file 2] provides the data set used in the present calculations along with derived calculations for reference values and explanations of how the data were obtained.

### Non-linear curve fitting

Excel's Solver feature (Tools menu, add-ins in Excel 2003; Office button/options/add-ins in Excel 2007) optimizes solutions in a particular cell by changing values in a range of cells that the user defines [17]. It is simple to use Solver to minimize the sum of squares of differences between observed data and theoretical values calculated from the model equation [see Additional File 1]. Parameter values are returned that provide best fit of an equation to data. The values reported at week S24 do not equal the theoretical asymptotes needed for Eq. 4. The asymptotes and optimal rate constants were calculated using Solver and also by a 3 point method described below (Eq. 6).

### Three point method for estimating rate parameters from loss ratios

A second method has been found for obtaining estimates for first order rate parameters and steady state values from data sets with at least three evenly spaced time points. In working with two 12 week time intervals, as is the case with the Minnesota data set, then the following relationship holds:

(5) $$k_{i}=-\frac{1}{12}\ln\left(\frac{M_{0\;}-\;M_{12}}{M_{12}\text{ - }M_{24}}\right)$$

In Eq. 5, k_i _is the first order rate constant (wk^-1^) that obtains for the *i*th subject or quantity (body mass, FM, etc.) from C12 until S24. In this case, M_0_, M_12 _and M_24 _are body masses at C12, S12 and S24. The number 12 in Eq. 5 represents the interval between measurements in weeks, and would need to be changed if a different interval was used. Once the value of k_i _is known, the projected steady state value (here, M_ss_) may be obtained:

(6) $$M_{ss}=\frac{\left(M_{0}\times e^{-k*24}-M_{24}\right)}{\left(e^{-k*24}-1\right)}$$

Note that the exponent, 24, is 2× the measurement interval. These two equations give specific solutions and may be solved quickly on scientific calculators or spreadsheets. It is not necessary to set up models and perform separate parameter estimates with Solver on each set of data. To solve Eq. 6 in Excel, the exponential function is represented as EXP(-k*24). This method provides values that are identical to the ones found using Solver.

## Results

### Approach to steady state for group mean body composition and function

**Figure 2:**
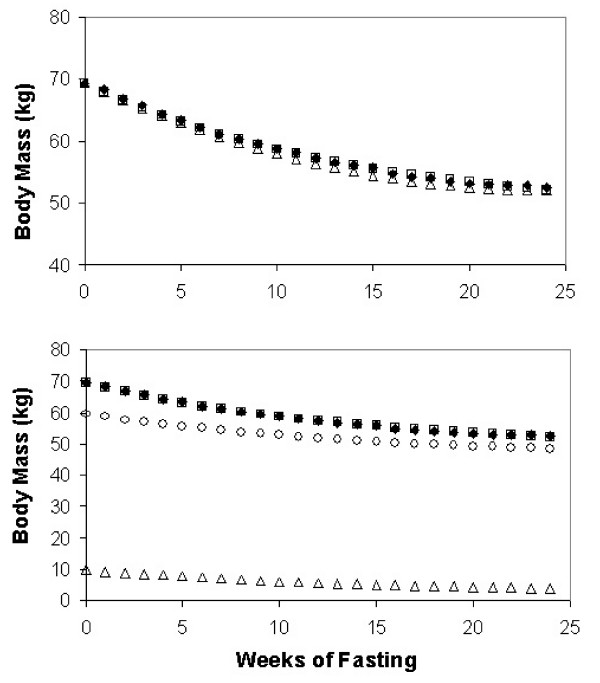
**Model behavior compared to predictive equation used by Keys**. Upper panel shows observed values for body mass (closed diamonds), values predicted by the Keys equation (open triangles), and results of fitting the data to a monoexponential approach to steady state (open squares). Lower panel shows solutions for fat mass (open triangles), fat free mass (open circles), calculated body mass (open squares) and observed body mass (filled diamonds) over 24 weeks of partial fasting.

The model of approach to steady state was first applied to the data for body mass by defining the last week of the control period (C12) as t = 0 and extending weekly values to t = 24 in the 24^th ^week of energy restriction (S24)(stippled bar in Figure 1) when mean energy intake was almost constant. During the starvation phase, mean body mass decreased 24% from 69.39 kg to 52.57 kg (Figure 1). Data for group mean body masses for all 24 weeks were fit to Eq. 4 with initial estimates of k = 0.1 wk^-1 ^and body mass at 24 wks = 52.57 kg for M_ss_. Excel's Solver (see supplement) returned a first order rate constant of k = 0.070 wk^-1 ^for the group average and a predicted steady state mass M_ss _= 48.2 kg. The correlation coefficient between observed and predicted weekly body masses was R^2 ^= 0.998, a slightly lower agreement with the observed data than the equation (Eq. 1) used in the original study (Figure 2, top), whose R^2 ^with observed data was 0.9985, with S.E.E. of 0.22 kg and 0.20 kg respectively. Unlike the method of approach to steady state, Keys' parabolic equation cannot be used to predict a steady state.

For FM and all other anthropometric and functional measures, the Minnesota study reported only 3 values at weeks C12, S12 and S24, respectively. The exponential model was fit to group means for 12 of these measures using the 3 point method (Table 1). Although non-linear fitting with Solver and the 3-point method give identical results when the same data are used, the 3 point parameters differed slightly from the ones calculated for all 25 data points. With the 3-point method, the value of k for body mass was 0.079 wk^-1 ^and the predicted asymptote was 49.57 kg. The 3-point method had the same correlation with observed weight as the 25-point method. The half life for change in body mass (0.693/k) was 8.8 weeks. If steady state were achieved after 5–6 half lives, this would have occurred in 44–53 weeks, which was beyond the duration of the study.

**Table 1:** Kinetic parameters for endpoints from the Minnesota starvation study based on group averages (n = 32).

**Quantity or Function**	**C12 mean**	**S12 mean**	**S24 mean**	**k (wk^-1^)^a^**	**Steady State**
Body mass (kg)	69.39	57.28	52.57	0.079	49.6
Fat mass (kg)	9.84	4.57	3.04	0.103	2.41
Fat free mass (kg)^b^	59.55	52.71	49.53	0.064	46.8
Body Mass Index^a ^(kg/m^2^)	21.7	17.92	16.44	0.078	15.5
Chest girth^b ^(cm)	89.28	83.69	82.61	0.137	82.4
Waist girth (cm)	78.08	71.23	70.7	0.213	70.7
Girth Sums^d ^(cm)	113.3	98	92.5	0.085	89.4
Active Tissue Mass^c ^(kg)	39.9	32.0	29.2	0.086	27.7
Red blood cells^c ^(kg)	2.74	2.11	1.99	0.138	1.96
REE^d ^(kcal/d)	1608	1100	994	0.131	966
Harvard work test	64.1	33.1	18.1	0.060	4.04
Hand dynamometer (kg)	58.2	47.2	41.8	0.059	36.6
N excretion (g/24 h)	13.17	8.12	7.42	0.165	7.31

Values for C12, the last week before energy restriction was imposed, were used as t = 0 in the model. S12 and S24 are the values recorded during the 12^th ^and 24^th ^weeks of energy restriction (t = 12 and 24 weeks, respectively).

^a^All parameter values were calculated from group mean data at C12, S12 and S24 by the 3 point method. Mean stature was 1.788 m.

^b^Fat free mass was calculated by subtracting measured fat mass from body mass.

^c^Active tissue mass equals body mass minus the sum of fat mass, bone mineral, and thiocyanate space. Group data for active tissue mass, and red blood cell mass are from Table 152, and mean chest girths are from Table 49 [1].

^d^Sum of girths of calf, arm and thigh. REE, resting energy expenditure.

FM declined at a more rapid rate (k = 0.103 wk^-1^) than total body mass. FFM was calculated by subtraction and fit well to a model with k = 0.064 wk^-1^. When summed, the predicted steady state values for FM and FFM equaled the predicted steady state body mass (Figure 2, lower panel). BMI declined at a rate similar to body mass, and approached a theoretical asymptote of 15.5 kg/m^2^. Active tissue mass, which is a component of FFM, declined at a faster apparent rate than FFM (which contains bone, brain and extracellular water). Waist girth changed at a faster rate (k = 0.213 wk^-1^) than any other measure. Parameters for REE, red blood cells, work and strength tests, and N excretion all fit the model well. In contrast, total plasma protein did not fit the exponential model well because the extent of change between S12 and S24 was similar to the change from C12 to S12 and the natural logarithm of the ratio was negative. REE changed at a faster rate than FFM, with which it is most closely correlated during stable weight. Initial REE was predicted by the following equation that gave best fit for the 32 subjects based on data at C12: 883+12.17 × FFM. Hence REE, as predicted from this equation, should have been 1525 and 1486 respectively at S12 and S24. Actual REE values were 1100 and 994, indicating that caloric restriction decreased energy expenditure per kg of FFM.

**Figure 3:**
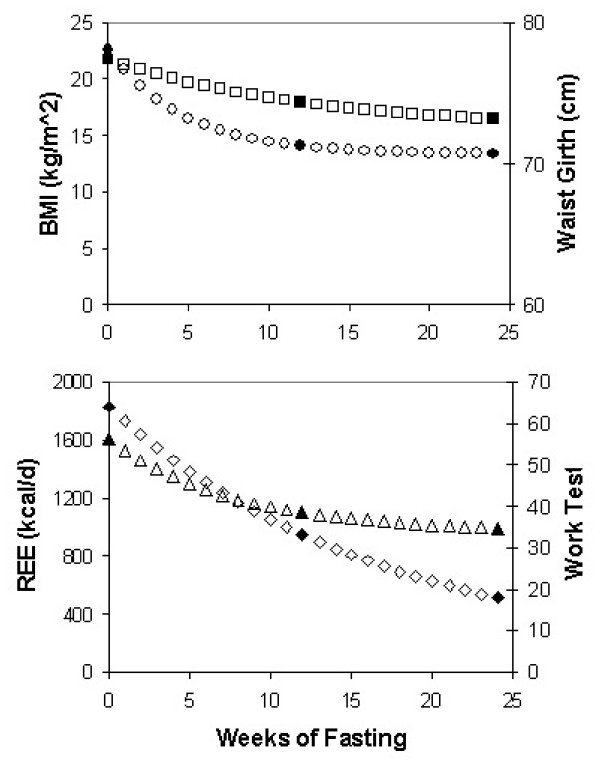
**Observed and theoretical values for study endpoints**. Top panel shows results for fitting data for BMI (squares) and waist girth (circles) to a monoexponential model for approach to steady state. Bottom panel shows results for resting energy expenditure (REE, triangles) and the Harvard work test (diamonds). The filled symbols represent the observed group means for each endpoint.

### Changes in girth and clinical tests

Most change in waist circumference occurred during the first 12 weeks of caloric restriction. A single exponential model with k = 0.213 wk^-1 ^and an asymptote of 70.7 cm fit the waist data well (Figure 3). Based on this value, the half time for change in waist girth in these lean male subjects was 3.25 wks and steady state would be attained in about 16–20 wks. A single exponential model was also sufficient to explain the change in sum of girths of the arm, thigh and calf (Table 1). The initial value was 113.3 cm and the asymptote was 89.4 cm with a first order rate constant of 0.085 wk^-1^. This equals a half-time of 8.1 wks. Limb girths were more resistant to change than abdominal girths, possibly because male subjects tend to store more fat in the abdomen than the limbs. Limb girths also reflected the slower loss of muscle mass.

The mean volume of the heart at systole decreased from 620 ± 86 mL at C12 to 514 ± 84 mL at S24. The change fit the model with a k value of 0.115 wk^-1 ^and a steady state volume of 506 mL. The pulse rate also decreased from 55.2 beats min^-1^, but no significant change occurred after S12, so the change could not be modeled. Urinary N loss per 24 h declined from an initial value of 13.17 g/24 h towards an asymptote of 7.3 g/24 h at a first-order rate of 0.165 wk^-1 ^and the results readily fit an exponential model (Table 1).

A single exponential model also fit the change in hand and back strength as assessed with a dynamometer (Table 1). Parameter values were: initial strength, 208 kg, asymptote, 129 kg, rate 0.06 wk^-1 ^(half time, 11.5 wk). Monoexponential models also described the results of a work capacity test and the Harvard fitness test (Figure 3, lower panel). Parameter values for the fitness test were: initial fitness score, 64.1; asymptote, 4.0; rate parameter, 0.06 wk^-1^; half time of 11.5 wks (80 days). The rates of change for these measures of strength and fitness were not statistically different from one another.

### Intersubject variability

Because the 3 point method employs the natural logarithm of a ratio, it is not appropriate to calculate means and standard deviations. Instead, Table 2 [see Additional file 3] shows the distributions for rate parameters (k) and predicted steady state values as a function of the change that occurred between week C12 and week S12 divided by the change from S12 to S24. We refer to this as the loss ratio. For an exponential model, the change during the first period must be greater than the change during the second period and neither change can be zero (see the Discussion section for an explanation).

### Intersubject comparisons

Table 3 [see Additional file 4] compares kinetic parameters for energy expenditure and body dimensions for the 2 most extreme subjects. Subject 5 was initially overweight (83.6 kg, BMI 25.4 kg/m^2^) at the beginning of the control period, and subject 127 was underweight (64.3 kg, BMI of 19.8 kg/m^2^) with estimated body fat of 8.7%. Subject 5's percent body fat at the end of the control period was 24.1% and his abdominal circumference of 91.8 cm was maximal for the group. Subject 5's weight loss goal was 29%, the maximum for the group. Differences between the two subjects included the speed with which REE declined during semi-starvation, the change of body mass, body fat, active tissue mass, waist girth, thigh girth, heart volume, and the Harvard fitness test. In contrast, rates of change in arm and calf girths were similar.

**Figure 4:**
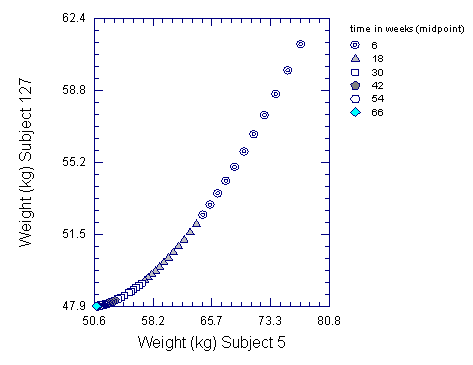
**Change in weight for a relatively lean subject compared to an overweight subject**. Comparisons between subjects were made based on best fit of the model to data from weeks C12 through S24 and then projecting through the midpoint of week 72. During the 24 weeks of the study, the model fit the data for subjects 5 and 127 with R^2 ^of 0.998 and 0.996, respectively.

As shown in Figure 4, the leaner subject 127's body mass dropped rapidly during the first twelve weeks and was within 97% of steady state at the end of S24. His rapid decline in REE equalled 648 kcal in the first 12 weeks. This brought him nearly into energy balance, and he lost only 0.6 kg of FM after S12. In contrast, overweight subject 5's body fat had declined to 10 kg by S12 and his REE of 1358 kcal d^-1 ^represented a decline of only 311 kcal d^-1^, which was less than half of the change for subject 127. Thus, subject 5's REE adapted more slowly to his energy deficit, and he continued to metabolize fat, losing 4.4 kg of FM between S12 and S24. The extent of adaptation is shown by the decline in REE per kg of FFM. For subject 5, the initial ratio was 27.2 kcal/kg, and this decreased to 24.2 at S12 and to 21.1 by S24. For subject 127, the initial ratio was 29.0 kcal/kg, and this decreased to 21.3 at S12 and 20.8 by S24. The most dramatic differences between these subjects were seen at S12.

From his body dimensions, we estimate subject 5's waist reference girth (see Methods section) to be 79.8 ± 1 cm. His initial waist girth of 91.8 cm was about 12 cm in excess of his expected waist girth, which he attained within 12 weeks. During this period, he lost 0.77 kg of body fat for every centimeter of waist reduction, a ratio typical for the group as a whole (5.3 kg of FM loss/6.9 cm of waist reduction).

Subject 127's initial waist girth equalled his waist reference girth of 73.4 ± 1 cm. Beyond a loss of 1–2 cm of residual abdominal fatness in the first two weeks of starvation, the remaining 4 cm of reduction in the waist girth by the end of 12 weeks must be attributed to loss of FFM or an upward shift of the umbilicus to a narrower waist level. Similarly, for Subject 5, waist girth was reduced to 77 cm, slightly below his waist reference girth of 79.8 cm, although body fat continued to decline. For both men, waist girth was the body dimension that reached steady state most rapidly. It is worth noting that Subject 5's relatively rapid reduction of bideltoid width and arm girth is a counterpart to his rapid loss of abdominal and upper body fat, while Subject 127's rapid loss of bicep muscle is more clearly demonstrated in the reduced bideltoid width rather than in the (unflexed) arm girth. The calf girth, on the contrary, was the most resistant to weight loss-related reduction. At 36 weeks, it was still 1% larger than predicted for steady state for both subjects. The low k value for calf girth loss of both subjects must be due to the stability of lower leg muscles at a site where neither subject has significant body fat.

## Discussion

Although any person who has attempted to lose weight has probably experienced the plateau effect on body mass, it is interesting that a simple model of approach to steady state seems to describe so many anthropometric and functional markers. The high correlation between observed and predicted results suggests that the model of approach to steady state, or plateau principle, is consistent with changes in waist girth, BMI, FM and FFM, REE, several anthropometric measures, heart volume, and tests of strength and work capacity that undergo adaptive change during periods of caloric restriction. Prior studies have shown that an exponential, asymptotic model is useful for analyzing changes in body composition during weight reduction [6] and weight gain during overfeeding [8], but other endpoints have not been analyzed. Our results show that the model can be applied not only to groups but also to individuals (Tables 1 & 3). The main reason that a plateau is achieved during partial fasting is the combined reduction in body mass and adjustments reducing REE per kg of FFM by over 25%. On average, a 32% reduction in REE must have taken place within the first 12 weeks of partial starvation.

Because waist girth and BMI are indices of abdominal obesity that are related to metabolic syndrome, the projected time course and magnitude of change are important clinical values that could be used to provide feedback to subjects in weight reduction programs. Unlike clinical measures that require blood sampling, individuals with access to spreadsheets could readily track these changes using measurements obtained at home. With an estimate of k and the steady state value for individuals or groups, one may use Eq. 3 to forecast body mass, waist girth, or other endpoints at future times if food intake and physical activity levels are maintained. The projected half-time for change in waist girth (3–4.4 weeks or 21–31 days) in lean men is substantially less than the 680 day half time for approach to steady state in human adipose fatty acid composition [18]. Although the rate parameters found in the present study do not necessarily apply to older, abdominally obese men or women, the results confirm that a consistent program of energy restriction and increased physical activity could produce significant risk reduction within 4–6 months. Published data for waist reduction during treatment of overweight adults with rimonabant (a cannabinoid receptor antagonist) [19] or treatment of adolescents with sibutramine (a serotonin reuptake inhibitor) [20] are consistent with an exponential loss that approaches a plateau. However, the regain of weight and waist girth during the crossover phase of the rimonabant study do not appear to be exponential even though a plateau is attained [19].

A number of sophisticated models have been developed to explain the change in energy partitioning during partial or total starvation [10,21-23]. Such models are very useful in stimulating mechanistic research, but are not necessarily suitable for individuals to use for data logging or classroom instruction. Our interest was specifically to determine whether a risk factor such as waist girth could usefully be modeled with spreadsheet software.

### Implementing the approach to steady state in spreadsheets

The approach to steady state or plateau principle is based on a very simple, mechanistic equation that expresses change over time as a function of rates of synthesis and turnover. Although we defined Eq. 3 in terms of mass rather than a rate of synthesis or formation, steady state is achieved for tissue mass when the synthesis rate (g/kg/time interval) equals the loss rate (which has the same units as the synthesis rate and equals existing mass times k, the fractional turnover rate [24]). The new steady state value can be calculated by dividing the production rate by the first order rate constant [25]. Especially for biological scientists, it is far easier to show the implications of Eq. 3 using spreadsheet software to generate graphs (see examples in the supplement) than it is to explain the concepts verbally.

Fitting Eq. 3 to observational data requires a method to estimate the rate parameter and asymptote. As shown in the supplement, the parameters can easily be obtained using the Solver utility in Microsoft Excel^®^. Even students with limited background in mathematics can grasp these concepts through hands-on tutorials in computer laboratories in which different scenarios can be tested by changing initial values and rate parameters. Alternatively, the 3 point method can be used to calculate rate parameters directly from the data. As the spreadsheet in the supplement should demonstrate, it is not difficult to apply the plateau principle in Excel to estimate parameters that fit model equations (theory) to observations [26,27]. It would be interesting to apply simple kinetic modeling to multi-compartmental models of human body composition [28-30].

A useful feature of the plateau principle is that the biological half life of any constituent to which it applies is related to the first-order elimination parameter (t_1/2 _= ln2/k_e _= 0.693/k_e_). The time required to attain 97% of a new steady state equals 5 half lives. At the level of tissues, turnover of specific proteins [25,31] and messenger RNA populations [7,32] has been modeled using the same principle. Using the same methods described in the present paper, a simple, two-compartmental model of gene expression has been implemented in Excel [24]. In pharmacology, the plateau principle provides a basis for drug dosing in which the amount of drug in the blood plasma can be predicted on the basis of amount administered, dosing interval, and the first order disposition rate from the blood plasma [33]. A technique called compartmental modeling is based on flow of materials that often follows simple exponential (first-order) kinetics, although more complex equations may be employed to reflect saturable or non-linear processes. Compartmental modeling has been used to analyze requirements for essential nutrients including amino acids [34-36], fatty acids [37,38], vitamins [39], and minerals [40,41] that cannot be synthesized in the human body. Recently, the principle of approach to steady state has been used to model plasma concentrations of 25-hydroxycholecalciferol [42] and ascorbic acid [43,44]. In both cases, model-based investigations suggest that the Recommended Dietary Intakes are somewhat lower than optimal.

### Notes on the 3 point method

The 3 point method for estimating rate parameters and asymptotes during an approach to steady state may be suitable for clinical studies of body composition or girth measurements when data are sparse. It can be applied to group means or to individual data, and it is easy to solve Eqs. 5 and 6 in a spreadsheet or with a scientific calculator. Although the Minnesota data were reported at 12 week intervals, the equations may be solved using other equally spaced numbers of days or weeks. The rate constants are defined in terms of the time frame used (per week or per day).

In using the three-point loss ratio analysis, we found that for data points observed over a period of 24 weeks, the exponential model yielded meaningful results wherever the loss ratio fell somewhere between 1.5 and 10 (k values between .034 and .19). A loss ratio of 1.5 would allow a plateau to be approached (5 half-lives) in 102 weeks, whereas a ratio of 10 would mean that the plateau is fully reached (7 half-lives) in 25 weeks. In Table 1, which uses the combined data of the 32 men, the underlying loss ratio falls within this range for all the variables except the waist girth. whose plateau is fully reached in less than 23 weeks. The model thus works well for the variables in Table 1.

The model works equally well for the different variables for a substantial majority of the individuals who participated in the study, as Table 2 indicates. The table also shows, however, that for a number of individuals and a number of variables, the loss ratio is not compatible with the exponential model. Where the loss ratio is 1 or less, the rate of change is either constant or positive and does not lead to a plateau. For reasons that are too closely linked to the specifics of the Minnesota study to explain here, the model fails most frequently for FM and FFM. Even where the loss ratio lies between 1 and 1.5, the predicted plateaus for body mass and FFM are unrealistically low in terms of the individuals' normal weight, while FM plateaus at a negative value. Conversely, for several individuals, there is extremely little or no loss or even a small gain between the first and the second loss interval, so that the loss ratio is extremely large or infinite. Here the model fails because the actual plateau must have been reached already during the first interval or long before the end of the second interval (as is true even for the group with respect to the waist girth). For several individuals, the same applies to REE and FM as well. (In Table 2, the individuals with such loss ratios have been arbitrarily included in the highest loss ratio category).

## Conclusion

Our analysis shows that during energy restriction, waist girth and several other anthropometric and physiological endpoints approach a plateau that is consistent with a simple and widely applicable kinetic model. For example, the steady state values predicted by Eq. 6 could represent targets for healthy waist girths or body mass normalized to frame size [13]. The model confirms that a realistic time frame for change in waist girth and other aspects of body composition must be expressed in weeks or months, and even then requires sustained changes in lifestyle as well as consideration of risk factors such as elevated blood pressure. The model could be applicable to changes in body composition during weight loss regimens, and possibly also to increases in mass or work capacity during strength training. As implemented in Microsoft Excel, the model can be solved using Solver or by the 3 point method. This software is available and familiar to most college faculty and students, and can be tested using a variety of public databases. We encourage colleagues to try these methods for model-based hypothesis testing.

## Competing interests

The authors declare that they have no competing interests.

## Authors' contributions

JLH initiated the modeling approach and implemented the model in Microsoft Excel. GH suggested applying the model to data from the Minnesota study and discovered the regular relationship between change during weeks 1–12 and 13–24. OH derived equations 5 and 6 to solve for model parameters and contributed extensively to discussions of the model. GH used these equations to provide all estimates of rate parameters and steady state values by means of the 3 point method. JLH and GH jointly prepared the figures and tables and wrote the manuscript. All authors read and approved the final manuscript.

## Supplementary Material

Additional file 1 Microsoft Excel^® ^spreadsheet for using Solver or the 3 point method to derive kinetic parameters for the model of approach to steady state. This spreadsheet contains numerical data from the Keys study and was written using Excel 2003. The worksheet with the tab labeled "Model Using Solver" gives instructions for how to obtain parameters for best fit of the data to the equation of approach to steady state. The worksheet labeled "Methods Compared" shows that the 3 point method gives the same estimates for rate parameters as Solver does when data are limited (compare results in cells G2 and G3 with cells L9 and L10). Readers may obtain the values for changes in heart volume and resting energy expenditure by placing the cursor in cell L9 and filling across to cells M9 and N9. Then do the same operation for cells L10 through N10. This operation solves equations 5 and 6 using data for the two endpoints. The method using Solver would be preferred for a study in which data have been collected at frequent intervals. Click here for file

Additional file 2 Microsoft Excel^® ^spreadsheet containing the complete primary data set and derived calculations for anthropometric and physiological endpoints in the starvation study. This file contains all measurements used to perform the calculations reported in our study. The first tab explains the variable names and data sources used. The second tab presents the compiled data organized as described in the first tab. The third tab explains how the original measurements were conducted, and the fourth tab explains how derived calculations were obtained. Click here for file

Additional file 3 Table 2. Distribution of kinetic parameters obtained by the 3 point method as a function of the loss ratio. Table 2 shows the variability among subjects that is obtained for rate constants and steady state values for different variables in the human starvation study. Click here for file

Additional file 4 Table 3. Comparison of rate parameters (k, wk^-1^) and steady state values (ss) for overweight subject 5 and underweight subject 127. Table 3 contrasts kinetic parameters for the two most extreme subjects in the human starvation study. Click here for file

## References

[B1] Keys A, Brozek J, Henschel A, Mickelsen O, Taylor HL (1950). The biology of human starvation.

[B2] Kleiber M (1975). The Fire of Life, An Introduction to Animal Energetics.

[B3] Benedict FG (1907). The influence of inanition on metabolism.

[B4] Howe PE, Mattill HA, Hawk PB (1912). Fasting studies. VI. Distribution of nitrogen during a fast of one hundred and seventeen days. J Biol Chem.

[B5] Lointier PH, Verdier PH, Verdier A (2003). A correlation method for weight loss after gastroplasty. Obes Surg.

[B6] Livingston EH, Sebastian JL, Huerta S, Yip I, Heber D (2001). Biexponential model for predicting weight loss after gastric surgery for obesity. J Surg Res.

[B7] Hargrove JL, Schmidt FH (1989). The role of mRNA and protein stability in gene expression. Faseb J.

[B8] Deriaz O, Tremblay A, Bouchard C (1993). Non linear weight gain with long term overfeeding in man. Obes Res.

[B9] Pitts GC, Bull LS (1977). Exercise, dietary obesity, and growth in the rat. Am J Physiol.

[B10] Hall KD (2006). Computational model of in vivo human energy metabolism during semistarvation and refeeding. Am J Physiol Endocrinol Metab.

[B11] Hall KD (2008). What is the required energy deficit per unit weight loss?. Int J Obes (Lond).

[B12] Hall KD, Bain HL, Chow CC (2007). How adaptations of substrate utilization regulate body composition. Int J Obes (Lond).

[B13] Heinz G, Ko GT, Peterson LJ (2005). Waist girth normalized to body build in obesity assessment. Asia Pac J Clin Nutr.

[B14] Heinz G, Peterson LJ, Johnson RW, Kerk CJ (2003). Exploring Relationships in Body Dimensions. J Statistics Ed.

[B15] Schimke RT, Sweeney EW, Berlin CM (1964). An analysis of the kinetics of rat liver tryptophan pyrrolase induction: the significance of both enzyme synthesis and degradation. Biochem Biophys Res Commun.

[B16] Rodgers JR, Johnson ML, Rosen JM (1985). Measurement of mRNA half-life as a function of hormonal treatment. Methods in Enzymology.

[B17] Harris DC (1998). Nonlinear least-squares curve fitting with Microsoft Excel Solver. J Chem Ed.

[B18] Dayton S, Hashimoto S, Dixon W, Pearce ML (1966). Composition of lipids in human serum and adipose tissue during prolonged feeding of a diet high in unsaturated fat. J Lipid Res.

[B19] Pi-Sunyer FX, Aronne LJ, Heshmati HM, Devin J, Rosenstock J (2006). Effect of rimonabant, a cannabinoid-1 receptor blocker, on weight and cardiometabolic risk factors in overweight or obese patients: RIO-North America: a randomized controlled trial. JAMA.

[B20] Berkowitz RI, Fujioka K, Daniels SR, Hoppin AG, Owen S, Perry AC, Sothern MS, Renz CL, Pirner MA, Walch JK, Jasinsky O, Hewkin AC, Blakesley VA (2006). Effects of sibutramine treatment in obese adolescents: a randomized trial. Ann Intern Med.

[B21] Flatt JP (2004). Carbohydrate-fat interactions and obesity examined by a two-compartment computer model. Obes Res.

[B22] Kozusko FP (2001). Body weight setpoint, metabolic adaption and human starvation. Bull Math Biol.

[B23] Song B, Thomas DM (2007). Dynamics of starvation in humans. J Math Biol.

[B24] Hargrove JL, Hulsey MG, Schmidt FH, Beale EG (1990). A computer program for modeling the kinetics of gene expression. Biotechniques.

[B25] Berlin CM, Schimke RT (1965). Influence of turnover rates on the responses of enzymes to cortisone. Mol Pharmacol.

[B26] Chamberlain J (2003). The use of spreadsheets for pharmacokinetic simulations. ScientificWorldJournal.

[B27] Johanson G, Naslund PH (1988). Spreadsheet programming – a new approach in physiologically based modeling of solvent toxicokinetics. Toxicol Lett.

[B28] Levitt DG, Heymsfield SB, Pierson RN, Shapses SA, Kral JG (2007). Physiological models of body composition and human obesity. Nutr Metab (Lond).

[B29] Wang Z, Heshka S, Pietrobelli A, Chen Z, Silva AM, Sardinha LB, Wang J, Gallager D, Heymsfield SB (2007). A new total body potassium method to estimate total body skeletal muscle mass in children. J Nutr.

[B30] Wu EX, Tang H, Tong C, Heymsfield SB, Vasselli JR (2008). In vivo MRI quantification of individual muscle and organ volumes for assessment of anabolic steroid growth effects. Steroids.

[B31] Schimke RT (1966). Studies on the roles of synthesis and degradation in the control of enzyme levels in animal tissues. Bull Soc Chim Biol (Paris).

[B32] Bhasi K, Forrest A, Ramanathan M (2005). SPLINDID: a semi-parametric, model-based method for obtaining transcription rates and gene regulation parameters from genomic and proteomic expression profiles. Bioinformatics.

[B33] Goldstein A, Aronow L, Kalman SM (1974). Principles of drug action: the basis of pharmacology.

[B34] Basile-Filho A, Beaumier L, El-Khoury AE, Yu YM, Kenneway M, Gleason RE, Young VR (1998). Twenty-four-hour L-[1-(13)C]tyrosine and L-[3,3-(2)H2]phenylalanine oral tracer studies at generous, intermediate, and low phenylalanine intakes to estimate aromatic amino acid requirements in adults. Am J Clin Nutr.

[B35] Millward DJ, Rivers JP (1988). The nutritional role of indispensable amino acids and the metabolic basis for their requirements. Eur J Clin Nutr.

[B36] Riazi R, Wykes LJ, Ball RO, Pencharz PB (2003). The total branched-chain amino acid requirement in young healthy adult men determined by indicator amino acid oxidation by use of L-[1-13C]phenylalanine. J Nutr.

[B37] Goyens PL, Spilker ME, Zock PL, Katan MB, Mensink RP (2005). Compartmental modeling to quantify alpha-linolenic acid conversion after longer term intake of multiple tracer boluses. J Lipid Res.

[B38] Pawlosky RJ, Hibbeln JR, Novotny JA, Salem N (2001). Physiological compartmental analysis of alpha-linolenic acid metabolism in adult humans. J Lipid Res.

[B39] Green MH, Uhl L, Green JB (1985). A multicompartmental model of vitamin A kinetics in rats with marginal liver vitamin A stores. J Lipid Res.

[B40] Lowe NM, Woodhouse LR, Sutherland B, Shames DM, Burri BJ, Abrams SA, Turnlund JR, Jackson MJ, King JC (2004). Kinetic parameters and plasma zinc concentration correlate well with net loss and gain of zinc from men. J Nutr.

[B41] Novotny JA, Turnlund JR (2006). Molybdenum kinetics in men differ during molybdenum depletion and repletion. J Nutr.

[B42] Heaney RP, Davies KM, Chen TC, Holick MF, Barger-Lux MJ (2003). Human serum 25-hydroxycholecalciferol response to extended oral dosing with cholecalciferol. Am J Clin Nutr.

[B43] Graumlich JF, Ludden TM, Conry-Cantilena C, Cantilena LR, Wang Y, Levine M (1997). Pharmacokinetic model of ascorbic acid in healthy male volunteers during depletion and repletion. Pharm Res.

[B44] Levine M, Wang Y, Padayatty SJ, Morrow J (2001). A new recommended dietary allowance of vitamin C for healthy young women. Proc Natl Acad Sci USA.

