# Supplementary material for: Modeling transitions in body composition: the approach to steady state for anthropometric measures and physiological functions in the Minnesota human starvation study
Source: Dyn Med. 2008 Oct 7;7:16. doi: 10.1186/1476-5918-7-16 (PMC2596786; doi:10.1186/1476-5918-7-16)
Supplement: Additional file 3 — Table 2. Distribution of kinetic parameters obtained by the 3 point method as a function of the loss ratio. Table 2 shows the variability among subjects that is obtained for rate constants and steady state values for different variables in the human starvation study. [file 1476-5918-7-16-S3.doc]

**Table 2. Distribution of kinetic parameters obtained by the 3 point method as a function of the loss ratio.**

| **Loss ratio**  **(C12-S12)/(S12-S24)** | **0 - 1** | **1 - 1.5** | **1.5 - 2** | **2 to 3** | **3 - 5.65** | **5.65 - 32** | **k for group**  **(wk^-1^)** |
| --- | --- | --- | --- | --- | --- | --- | --- |
| **REE (kcal/d)** |  |  |  |  |  |  |  |
| No.Subjects | 0 | 2 | 0 | 6 | 13 | 8 |  |
| k (wk^-1^) |  | .01-.03 |  | .062-.089 | .095-.131 | .145-.270 | 0.131 |
| steady state |  | n.s. |  | 839-961 | 815-1155 | 823-1150 |  |
| **Body Mass (kg)** |  |  |  |  |  |  |  |
| No.Subjects | 0 | 0 | 5 | 20 | 5 | 2 |  |
| k (wk^-1^) |  |  | .035 - .052 | .064 - .088 | .099 - .138 | .167 -.234 | 0.0787 |
| steady state |  |  | 38.6 - 48.6 | 45 - 55.2 | 46.4 - 57.4 | 48.8 - 53.4 |  |
| steady state/reference weight |  |  | 60 - 63% | 68 - 78% | 72 - 79% | 74 - 78% |  |
| **Girth Sum (cm)** |  |  |  |  |  |  |  |
| No.Subjects | 0 | 1 | 5 | 13 | 8 | 5 |  |
| k (wk^-1^) |  | 0.0267 | .038 - .057 | .063 - .089 | .097 - .138 | .149 - .20 | 0.085 |
| steady state |  | n.s. | 69.8 - 86.8 | 83 - 89 | 87.2 - 97.2 | 87.2 - 95.7 |  |
| **Calf Girth (cm)** |  |  |  |  |  |  |  |
| No.Subjects | 0 | 4 | 5 | 11 | 7 | 5 |  |
| k (wk^-1^) |  | .005 - .029 | .0035 - .053 | .061 - .083 | .093 - .143 | .147 - .172 | 0.0786 |
| steady state |  | n.s. | 29 - 33 | 28.7 - 34 | 31 - 35.2 | 31.7 - 35.5 |  |
| **Waist Girth (cm)** |  |  |  |  |  |  |  |
| No.Subjects | 0 | 1 | 2 | 1 | 11 | 16 |  |
| k (wk^-1^) |  | 0.003 | .036 - .037 | 0.068 | .098 - .143 | .145 - .199 | 0.213 |
| steady state |  | n.s. | 61.4 - 62 | 64.2 | 64.2 - 75.7 | 66.8 - 76.7 |  |
| **Fat Mass (kg)** |  |  |  |  |  |  |  |
| No.Subjects | 4 | 4 | 3 | 2 | 8 | 9 |  |
| k (wk^-1^) | -.029-.001 | .003-.026 | .041 - .045 | .066 - .068 | .098-.125 | .148 - .207 | 0.103 |
| steady state | n.s. | n.s. | n.s. to 1.3 | n.s. - 1.58 | 1.7 -4.4 | .7 - 3.8 |  |
| **Fat Free Mass (kg)** |  |  |  |  |  |  |  |
| No.Subjects | 5 | 7 | 3 | 3 | 7 | 6 |  |
| k (wk^-1^) | -.028 - .005 | .002 - .023 | .035-.055 | .059 - .079 | .100 - .142 | .152 - .20 | 0.0638 |
| steady state | n.s. | n.s. - 39.6 | 40.4 - 47.9 | 45.4 - 49 | 45.2 - 51.6 | 46.9 - 48.3 |  |

The loss ratio is defined as the change in a specific measurement from week C12 to S12 divided by the change from week S12 to S24.
