# Supplementary material for: Modeling transitions in body composition: the approach to steady state for anthropometric measures and physiological functions in the Minnesota human starvation study
Source: Dyn Med. 2008 Oct 7;7:16. doi: 10.1186/1476-5918-7-16 (PMC2596786; doi:10.1186/1476-5918-7-16)
Supplement: Additional file 4 — Table 3. Comparison of rate parameters (k, wk-1) and steady state values (ss) for overweight subject 5 and underweight subject 127. Table 3 contrasts kinetic parameters for the two most extreme subjects in the human starvation study. [file 1476-5918-7-16-S4.doc]

**Table 3. Comparison of rate parameters (k, wk^-1^) and steady state values (ss) for overweight subject 5**

and underweight subject 127

|  | **Subject 5** | | | | | **Subject 127** | | | | |
| --- | --- | --- | --- | --- | --- | --- | --- | --- | --- | --- |
|  | **C12** | **S12** | **S24** | **k** | **ss** | **C12** | **S12** | **S24** | **k** | **ss** |
| REE^a^ (kcal d^-1^) | 1669 | 1358 | 1099 | 0.0277 | 495 | 1704 | 1056 | 976.8 | 0.175 | 966 |
| Body Mass (kg) | 80.8 | 64.6 | 57.1 | 0.0642 | 50.6 | 64.2 | 52.5 | 49.2 | 0.1055 | 47.9 |
| Body Fat (kg) | 19.4 | 9.5 | 5.1 | 0.0676 | 1.58 | 5.6 | 2.9 | 2.3 | 0.125 | 2.1 |
| Active Tissue (kg) | 41 | 34.7 | 31.6 | 0.0591 | 28.6 | 39.1 | 30.1 | 27.4 | 0.1 | 26.2 |
| Waist Girth (cm) | 91.8 | 78.8 | 77.1 | 0.17 | 76.8 | 72.4 | 66.9 | 66.5 | 0.2184 | 66.3 |
| Calf Girth (cm) | 39.6 | 36.2 | 34.4 | 0.053 | 32.4 | 37 | 33.6 | 31.5 | 0.074 | 30.4 |
| Arm Girth (cm) | 32.4 | 26.2 | 24 | 0.0863 | 22.8 | 28.2 | 24 | 22.2 | 0.0817 | 21.1 |
| Thigh Girth (cm) | 51.8 | 44.5 | 40.8 | 0.0566 | 37 | 44 | 36.7 | 34.6 | 0.1033 | 33.75 |
| CAT^a^ Girth Sum (cm) | 123.8 | 106.9 | 99.2 | 0.0655 | 92.8 | 109.8 | 94.3 | 89 | 0.0894 | 86.2 |
| Bideltoid Width (cm) | 45.6 | 42.6 | 41.5 | 0.0863 | 40.9 | 41.7 | 37.5 | 37 | 0.177 | 36.9 |
| Harvard Fitness Test | 26 | 22 | 19 | 0.024 | 10.01 | 78 | 36 | 19 | 0.0754 | 7.45 |

^a^REE, resting energy expenditure. CAT, calf arm and thigh girths
